# Self or (M)other? Infants’ Sensitivity to Bodily Overlap With Their Mother Reflects Their Dyadic Coordination

**DOI:** 10.1111/cdev.13361

**Published:** 2020-04-01

**Authors:** Lara Maister, Lilla Hodossy, Manos Tsakiris, Jeanne L. Shinskey

**Affiliations:** ^1^ Royal Holloway University of London; ^2^ School of Psychology Bangor University; ^3^ The Warburg Institute University of London

## Abstract

Adults experience greater self‐other bodily overlap in romantic than platonic relationships. One of the closest relationships is between mother and infant, yet little is known about their mutual bodily representations. This study measured infants’ sensitivity to bodily overlap with their mother. Twenty‐one 6‐ to 8‐month‐olds watched their mother’s face or a stranger’s face being stroked synchronously versus asynchronously with their own face. Infants preferred synchrony only when viewing their mother, not when viewing the stranger. Infants who strongly preferred synchrony with their mother also experienced less coordination with her in naturalistic interactions. Infants thus appear sensitive to bodily overlap with their mother, and this overlap reflects dyadic coordination, supporting theoretical accounts of intersubjectivity in the development of the bodily self.

From birth, infants start to develop a sense of self, and in parallel, a way of relating to others. It is crucial that they integrate multiple sensory and motor signals for both self‐development and the development of self‐other relations. As past literature suggests, such processes of integration scaffold the social self (Fonagy, Gergely, & Target, 2007), although integration of perfectly contingent signals may play a different role from that of *imperfect* social contingencies. Whereas the former may be crucial for constructing body awareness, the latter may be important for constructing appropriate self‐other relations. The present study investigates the relation between these two types of integration in infancy, as a means of understanding the interdependence between body awareness and social interactions.

Early in life, infants initially may have only a rudimentary distinction between representations of their bodies and those of other people (Rochat, 2003). Through self‐exploration, they learn what belongs to their body and what does not, and furthermore what objects and individuals in the environment they can directly control. Their perception of contingent sensorimotor information plays a crucial role in this process (Bahrick & Watson, 1985; Filippetti, Johnson, Lloyd‐Fox, Dragovic, & Farroni, 2013; Gergely & Watson, 1999; Neisser, 1988; Rochat & Morgan, 1998). The visual‐proprioceptive and visual‐tactile stimulation that is perfectly contingent is uniquely “self‐specifying.” When infants see something moving or being touched at exactly the same time that they experience themselves moving or being touched, they are most likely observing either their own body or something closely related to it. Infants can use such contingencies to identify self‐performed actions as early as 3 months of age (Rochat & Morgan, 1998). For example, 5‐month‐olds discriminate between a perfectly contingent live display of their own leg motion and a noncontingent display of the self or a peer by looking longer at the noncontingent display (Bahrick & Watson, 1985). Infants’ sensitivity to such contingencies supports their developing representation of the bodily self and its distinction from other objects in the environment (Gergely & Watson, 1996, 1999).

More recent evidence shows that visual‐tactile synchrony contributes to body perception from birth (Filippetti, Farroni, & Johnson, 2016; Filippetti et al., 2013; Zmyj, Jank, Schütz‐Bosbach, & Daum, 2011). For example, newborns only 24‐hr old prefer to look at a video of another infant’s face being stroked in perfect synchrony, rather than asynchrony, with their own face (Filippetti et al., 2013). Likewise, both 7‐ and 10‐month‐old infants prefer to look at a video of a doll’s legs being stroked synchronously, rather than asynchronously, with their own legs (Zmyj et al., 2011). Importantly, in both studies mentioned, infants preferred perfect contingencies only with the body‐related stimuli of upright faces and dolls’ legs and not with control stimuli of inverted faces or wooden blocks. This pattern suggests that, rather than being an effect of a general preference for redundant sensory information, infants’ preference for perfect visual‐tactile contingencies may play a specific role in body representation. This comparison of sensitivity to synchronous versus asynchronous visual‐tactile stimulation was inspired by work showing that such synchrony induces illusions of body ownership in adults. For example, the rubber hand illusion (Botvinick & Cohen, 1998) happens when participants who view a prosthetic hand being touched in synchrony with their own hand, hidden from their view, subjectively report an experience of ownership over the prosthetic hand. These effects testify to the self‐specifying quality of perfect multisensory (i.e., visual‐tactile) contingency information.

In contrast, multisensory or sensorimotor events that are closely related in time but not perfectly synchronous are not uniquely self‐specifying, but instead suggest an interaction with another person or an object. In social exchanges with another person, the partner’s behavior is often causally responsive, but not perfectly synchronous, with the infant’s movements and expressions (e.g., Feldman, Greenbaum, Yirmiya, & Mayes, 1996). Infants find such imperfect but responsive contingencies highly engaging (Feldman, 2007; Markova & Legerstee, 2006) and become distressed when they are withdrawn, as evidenced by the still‐face paradigm (Tronick, Als, Adamson, Wise, & Brazelton, 1978).

Infants’ desire for these imperfect social contingencies is evident from how often they engage in coordinated face‐to‐face interactions with their parents (Feldman, 2007). From an early age, parents and infants engage in short and intense face‐to‐face interactions characterized by one member of the dyad responding to the other’s behavioral states of affect, attention, or arousal in a contingent turn‐taking pattern (e.g., Tronick, Als, & Brazelton, 1980). The effects of such interactions on infants’ social, cognitive, and emotional development are well‐documented (Feldman, 2007; Jaffe et al., 2001). Well‐coordinated parent–infant interactions are linked with the development of early joint attention, self‐regulation during the still‐face paradigm (Moore & Calkins, 2004), as well as symbol formation, IQ, and empathy in later childhood (Feldman et al., 1996; Harrist & Waugh, 2002). They are also linked with more secure attachment patterns (Beebe et al., 2010; De Wolff & Van Ijzendoorn, 1997; Isabella & Belsky, 1991), and the development of bodily self‐awareness (Fotopoulou & Tsakiris, 2017; Harel, Oppenheim, Tirosh, & Gini, 1999). Conversely, discordant mother–infant interactive contingencies can reflect difficulties associated with conditions such as preterm birth or maternal depression (Beebe et al., 2008; Feldman, 2007). Crucially, these face‐to‐face interactions provide sensorimotor stimulation to the infant that is *imperfectly contingent* with the infant’s own behavior, both in temporality and content, signaling to the infant that they are interacting with another person in a social context, rather than stimulating themselves (Bahrick & Watson, 1985). Detecting these imperfect contingencies is fundamental for the infant’s further development of a self‐other distinction (Bigelow, 1998; Gergely & Watson, 1999).

The extent to which representations of self and other are distinct or overlapping during social exchanges is crucial for the way that individuals process social information (Maister & Tsakiris, 2015). The concept of self‐other bodily overlap refers to the remapping of another’s bodily state onto the self, such that observing the other’s bodily experiences (e.g., movement, touch, pain, or emotion) results in a sharing or “resonance” with their experience as if it were one’s own (Keysers, Kaas, & Gazzola, 2010). The extent to which representations of self and other overlap reflects a continuum ranging from almost complete overlap at one end to clear self‐other distinction at the other end, where the other’s experiences are represented objectively as separate from those of the self. Both behavioral and neural investigations in adults confirm that social factors modulate the extent of self‐other bodily overlap versus differentiation. If an individual belongs to one’s own racial or social group, there is a greater “resonance” with their actions (Molnar‐Szakacs, Wu, Robles, & Iacoboni, 2007), tactile experiences (Serino, Giovagnoli, & Làdavas, 2009), and pain (Azevedo et al., 2013).

However, until recently, self‐other overlap was investigated only between unknown individuals and for broad social distinctions or brief impersonal interactions. Recently, Maister and Tsakiris (2016) extended this line of research to consider self‐other overlap or differentiation within intimate relationships, to find that dyads in a romantic relationship showed greater overlap in their embodied representations of each other’s movements than dyads in a platonic relationship. Specifically, romantic partners engaged in more automatic imitation of each other’s motor actions, which is known to increase affiliation (Chartrand & Bargh, 1999). Remarkably, the extent of bodily overlap was predicted by the quality of attachment. Individuals with more anxious attachment styles had more overlap between their representations of self and partner (Note that the extent of overlap that one member of a dyad experiences is independent of that experienced by the other member. Thus, one individual may have highly overlapping self‐ and partner representations, but their partner may represent themselves as very distinct and separate). These findings suggest that bodily overlap plays a key role in intimate relationships in adulthood, characterized by quantitative differences compared to other social relationships (e.g., friendship) and qualitative aspects of the relationship.

However, one of the most crucial and formative relationships across an individual’s lifespan is not initiated during adulthood, but is present from birth. The relationship between infant and mother is often one of the closest and most intimate relationships humans experience, and is fundamental for survival (Ainsworth, 1969; Broad, Curley, & Keverne, 2006; Fotopoulou & Tsakiris, 2017). Might infants’ relationship with their mother be characterized by high bodily overlap, as romantic relationships are in adulthood? Although the mechanisms of attachment may not be equivalent in these two kinds of relationships, infant–mother relationships share similar features with adult romantic relationships, such as having intimate bodily contact and feeling secure when the other is nearby and responsive (Hazan & Shaver, 1987). High bodily overlap might thus occur between infants and their mothers, as compared to others, and such overlap might play a unique role in the infant’s self‐ and social‐development. To the best of our knowledge, there is no work to date that explores bodily overlap between infants and mothers.

It is also unknown whether qualitative aspects of the infant–mother relationship affect their shared body representations, in an analogous way that they do in adults’ romantic relationships (Maister & Tsakiris, 2016). The structured, turn‐taking reciprocity inherent to natural parent–infant interactions provides imperfect contingencies that should support the development of a clear self‐other distinction (Gergely, Koos, & Watson, 2010). Thus, infants who experience well‐coordinated interactions with their mothers might have a more distinct, differentiated self‐other boundary, and therefore show a weaker preference for perfect contingencies with their mother. Conversely, infants who experience poorly coordinated interactions such as inconsistency or under‐involvement (predictors of later insecure attachment; e.g., Isabella & Belsky, 1991) may have unusually high levels of bodily overlap, and thus very low levels of self‐differentiation from the mother and a drive toward self‐specifying, perfectly contingent information in her presence. Recent findings suggest that infants who experience less‐coordinated interactions have stronger preferences for perfectly contingent stimulation in their mother’s presence. For example, 3‐month‐olds of less affectively attuned mothers gaze more when their mothers imitate them than when they interact naturally, whereas infants of more highly attuned mothers do the reverse (Markova & Legerstee, 2006). Likewise, 6‐month‐olds whose parents reported them as having more social interaction difficulties showed weaker self‐other discrimination by gazing more at a live video of their own perfectly contingent leg activity than at a delayed video of their noncontingent leg activity (Zmyj & Klein‐Radukic, 2015). These findings imply that individual differences in early social interaction affect infants’ ability to distinguish their own body from that of others. However, no research to date has addressed whether the quality of interactions between mother and infant affect their bodily overlap.

## The Current Study

The current study had two aims. The first was to assess infants’ sensitivity to bodily overlap with their mothers, using an infant version of synchronous visual‐tactile methods known to induce bodily overlap in adults and children (Botvinick & Cohen, 1998; Cowie, Makin, & Bremner, 2013). We hypothesized that infants’ bodily self‐representation would be less clearly differentiated from their mother than from strangers, that is, a greater self‐other overlap with the mother. Therefore, we expected infants to have a greater drive to seek self‐specifying, perfect contingencies when viewing their mother rather than a stranger. To test this hypothesis, we measured infants’ looking preference between paired videos of their mother’s face being touched synchronously versus asynchronously with the infant’s own face and compared it to their looking preference between paired videos of an unfamiliar woman’s face being touched synchronously versus asynchronously with their own. In addition to expecting that infants would look longer at perfectly synchronous than asynchronous contingencies, replicating previous studies (Filippetti et al., 2016; Zmyj et al., 2011), we predicted that infants would prefer these perfectly synchronous contingencies more when viewing their mother than when viewing a stranger. This finding would be consistent with the possibility that infants subjectively perceive greater self‐other overlap with the bodily experiences of their mother versus a stranger in a similar way that adults in romantic relationships experience more overlap than those in platonic relationships (Maister & Tsakiris, 2016).

The second aim was to relate infants’ looking preferences to patterns of naturalistic interactions with their mothers. We hypothesized that individual differences in infants’ preference for perfect contingency with their mother would be related to relationship quality, as indicated by the type of social interactions that the infant experiences with their mother (Feldman, 2007). Specifically, we expected that the degree of infants’ preference for viewing the synchronous video of their mother would correlate inversely with the degree of coordination during naturalistic face‐to‐face interactions with her. This prediction is based on theory and evidence described above that infants’ preference for imitative or perfect contingencies is related to less optimal parent–infant interactions (Beebe et al., 2008; Gergely et al., 2010; Jaffe et al., 2001; Markova & Legerstee, 2006; Zmyj & Klein‐Radukic, 2015), and that the degree of bodily overlap between individuals in a close relationship is related to qualitative aspects of that relationship (Maister & Tsakiris, 2016). Confirming this prediction would support the idea that experience with well‐coordinated but imperfect social contingencies helps infants develop the distinction between self and other (Bahrick & Watson, 1985). To test this hypothesis, we measured how coordinated mother–infant dyads were in their affective and attentive states during natural face‐to‐face interactions (Feldman et al., 1996; Gottman, 1981; Tronick et al., 1980), and related this coordination to infants’ looking preference on the visual‐tactile task. We focused on two key areas of nonverbal coordination; affect and attention (following Feldman, 2014). Affect was deconstructed into two constituent elements, arousal and valence, following the two‐dimensional or circumplex model of affective space (Russell, 1980). This model describes the structure of affective experience and provides a key framework for interpreting infants’ responses to affective stimuli (e.g., Stifter & Moyer, 1991).

## Method

### Participants

Twenty‐one full‐term typically developing infants between the ages of 6 and 8 months old (*M*_AGE_ = 6.6 months, *SD* = 0.55) participated in the final sample, along with their mothers. We chose this age because infants have been shown to prefer synchrony to asynchrony in visual‐tactile tasks at 5, 7, and 10 months of age (Filippetti et al., 2016; Zmyj et al., 2011) and because of the abundance of research into face‐to‐face parent–infant interactions in the second half of the first year (Feldman, 2007). We based a priori sample size on effect sizes calculated from published data for visual‐tactile synchrony effects in newborns (Filippetti et al., 2013) and 5‐month‐olds (Filippetti et al., 2016). A sample of 16 yields 95% power to detect a medium effect size of 0.5 in a 2 × 2 within‐participants design, according to G*Power (version 3.1.9.2; Faul, Erdfelder, Lang, & Buchner, 2007). Infants were recruited from a database of families from middle‐class counties in the southeastern UK who had registered their interest in research participation. Their race was 78% White, 11% Asian, and 4% more than one race. Data were collected from February 2015 through April 2016.

### Procedure

Each mother and infant attended two sessions, approximately 7 days apart. Mothers and infants arrived for the first testing session and were introduced to the experimenters and testing room. The main purpose of the first visit was to record video stimuli featuring the infant’s mother to be used in the bodily overlap task during the second visit, as well as to record a naturalistic face‐to‐face interaction that could be later coded for behavioral coordination. Once the infant was comfortable with the experimenters and the testing environment, the mother sat for the stimulus recording while the experimenter interacted with the infant in the same room. During this interaction, the experimenter familiarized the infant with being stroked on the face so that the infant would not find the sensation to be novel and distracting during the bodily overlap task at the second visit the following week. She did this by occasionally delivering a stroke on the infant’s cheek with a soft paintbrush, until the infant ceased orienting to the brush when it touched their face. After this, the infant was placed in a highchair, the cameras were repositioned and the face‐to‐face interaction commenced. In the second visit, infants completed the bodily overlap task using the prerecorded stimuli from their mother, and recordings from an unfamiliar woman. At the end of the second session, mothers were reimbursed for travel expenses, debriefed and given a small present as a token of thanks.

### Mother–Infant Bodily Overlap Task

#### Mother Stimulus Recording

Mothers were seated in front of a Panasonic Leica HC‐X920 full HD video camera positioned 60 cm away from them at head height. They were instructed to gaze directly at the camera with a neutral expression for the duration of the recording. An experimenter was positioned to the mother’s right, and delivered brief strokes with a soft paint brush (2.5 cm width) from the cheekbone to just above the jaw. The strokes were delivered at a frequency of one per 6 s, with each touch lasting approximately 1 s. The raw video footage was cropped around the face, leaving the brush present on the screen only for the time of stroking and when it dynamically approached or moved away from the face. The brush disappeared from view between strokes.

After the session, the videos were processed to adjust luminance and contrast to match the recording from an unfamiliar age‐ and race‐matched woman, selected from the database of other mothers. This unfamiliar woman was also selected to match the infant’s mother, as far as possible, with regards to hair color and whether they wore glasses. A different unfamiliar woman was used for each infant and remained the same throughout their task. Videos were cut into 21‐s clips that each featured three strokes. Each video included an initial baseline period where the mother gazed into the camera for 4 s, before the three strokes commenced.

#### Task Setup and Design

The Mother–Infant Bodily Overlap (MIBO) task was carried out at the beginning of the second testing session, after a period of settling the infant in the testing room. During the task, the infant sat in a highchair positioned 60 cm from the monitor, in front of a 74‐cm computer screen. An experimenter was positioned to the left and slightly behind the infant, in a position that allowed delivery of gentle touches to the infant’s face without drawing the infant’s gaze away from the screen. The mother sat directly behind the infant and was asked to avoid speaking or interacting with the infant during the task. A concealed video camera was positioned just below the computer screen in a central position, allowing infants’ looking times to each side of the screen to be later coded.

**Figure 1:**
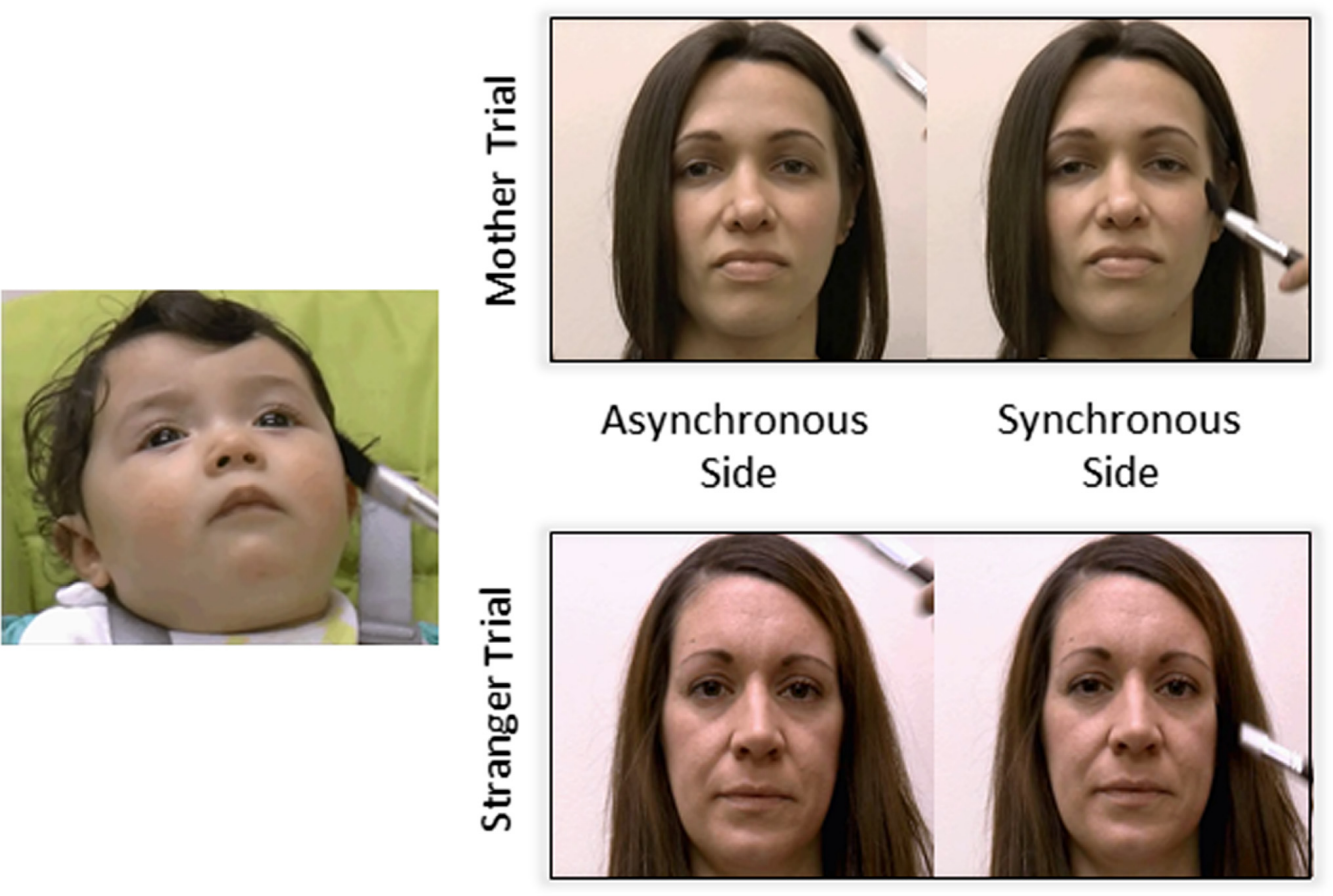
Diagram showing method for Mother–Infant Bodily Overlap task, which had a 2 × 2 Stimulation (synchronous vs. asynchronous) × Identity (mother vs. stranger) design. In each trial, the infant viewed two videos side‐by‐side, either both featuring the mother or both featuring an unfamiliar woman. The infant’s face was stroked in synchrony with one video and asynchrony with the other. Looking times to the two side‐by‐side videos were measured for both “mother” and “stranger” trials. [Color figure can be viewed at wileyonlinelibrary.com]

For each trial, infants viewed two videos simultaneously side‐by‐side. The videos were identical, both showing either the infant’s mother or an unfamiliar woman being touched on the cheek. Crucially, however, the timing of one video was delayed by 3 s relative to the other video (see Filippetti et al., 2016), so that the cheek touches were not synchronized. During each trial, the experimenter stroked the infant’s corresponding cheek in synchrony with one of the videos, which was asynchronous with the other video. Thus, in each trial the infant saw a synchronous and an asynchronous stroking video side‐by‐side. The side (left or right) on which the synchronous video appeared, and which video (synchronous or asynchronous) showed the first touch, was counterbalanced between trials. The identity of the individual in the video, either the mother or the stranger, was randomized across trials. Therefore, the task has a 2 × 2 Stimulation (synchronous vs. asynchronous) × Identity (mother vs. stranger) design (see Figure 1).

To allow infants time to orient to the screen before the first visual‐tactile stimulus was delivered, and to allow the experimenter to synchronize stimulation correctly, each video included a 4‐s baseline period depicting a static face prior to the tactile stimulation onset. This also enabled the assessment of any baseline differences in looking time to the two facial identities, which are independent of the tactile stimulation. Between each trial, infants were shown an attention‐getter of a short 2‐s audio‐visual clip of an engaging, nonsocial object presented centrally on the screen (e.g., a car, bouncing ball, or steam train). When the infant looked away from the screen for longer than 6 s (i.e., the duration of one stroke cycle), a second experimenter, who was positioned behind a curtain partition, initiated a longer nonsocial video clip with music to give the infant a short break and an incentive to reorient to the screen. Once the infant looked back at the screen, the second experimenter started the next trial. Trials continued until the infant looked away from the screen for longer than 6 s for three consecutive trials (as timed by the second experimenter viewing the infant’s gaze via live camera), became too tired or fussy to continue, or reached the maximum number of 20 trials.

#### Coding

Videos of infants’ looking during the MIBO task were scored by trained coders. For each trial, individual looking times were coded frame by frame for each of the two side‐by‐side videos, beginning from the first stroke that occurred when the infant was looking at the screen, until the end of the trial. The looking times were coded in this way because our stimuli were visual‐tactile, rather than just visual. Therefore, the onset of attention to our visual‐tactile stimulus was defined when the infant was experiencing both the visual and tactile components of the stimulus. The primary coder was blind to the condition (mother vs. stranger) while coding looking times to the left and right side of the screen. A smaller sample of recordings (10%) were independently scored by a second coder blind to the hypotheses behind the study. Inter‐rater reliability was good, intraclass correlation coefficient (ICC; 2,1) = .874, 95% CI [.369, .979].

### Behavioral Coordination

#### Task Setup

The infant was seated in a highchair facing their mother who sat in a low chair 50 cm in front of them. Two unobtrusive cameras were located in the room, one focused on the infant and one on the mother. The cameras first recorded an auditory signal to be used for later temporal synchronization. Once the infant was settled in the highchair, the mother was asked to interact with her infant in a normal way “as she would do at home” for 5 m, following the method of Feldman, Greenbaum, and Yirmiya (1999) for examining mother–infant affect synchrony. After setting the cameras to record, the experimenters left the room to ensure the situation was as natural as possible.

#### Coding

Videos were analyzed offline for affective and attentive synchronicity by five trained independent coders who were unaware of the hypotheses of the study (Feldman, 2014). Videos were separated into clips showing only the mother and clips showing only the infant, so that they were coded independently. Before coding, the first and last minute of every interaction session was removed, as according to previous research, the most pronounced social interaction occurs between the second and fifth minute in a 5‐m naturalistic play session (Feldman, 2003).

To code the videos, continuous ratings from a number of independent coders were recorded by Dual/Continuous Axis Rating and Media Annotation (DARMA/CARMA) software specifically developed for real‐time, naturalistic coding of affective and interpersonal processes (Girard & Wright, 2018). Affect was measured on the two dimensions of valence (unpleasant to pleasant) and arousal (low energy to high energy) based on the affective circumplex model (Russell, 1980) using DARMA. Attention was measured on a single dimension capturing gaze toward the partner’s face using CARMA. This program continuously records the position of the coder’s joystick on one or two dimensions (for attention, or affect valence and arousal, respectively) with a previously specified sampling rate (i.e., 1/s in the present study). This method of coding is continuous, and therefore captures dynamic changes across time that are less well‐characterized by discrete coding categories. However, it is potentially more susceptible to individual differences in how each coder utilized and conceptualized the rating scale. This is why a larger number of coders are generally used for this approach to ensure reliability.

Coders rated each video twice. First, in a single viewing, coders rated the valence and arousal of affect independently but simultaneously by moving the joystick in a two‐dimensional space known as a bi‐dimensional affective circumplex (Russell, 1980), with valence on the *x*‐axis and arousal on the *y*‐axis with a scale from −100 to +100. For example, they rated a momentary expression of highly aroused positive affect by placing the joystick in the upper right quadrant. If the expression then changed to a low‐arousal, negative state, the rater moved the joystick toward the lower left‐hand quadrant. More specifically, to score valence, raters used the lower half of the scale (−100 to 0) if they judged the individual’s affective expression to be negative, giving lower scores for more intense expressions. They used the upper half of the scale (0 to 100) if they judged the affective expression to be positive, giving higher scores for more intense expressions. To score arousal, raters gave lower scores if they judged the affective expression to be relaxed or low in energy and higher scores if they judged it as lively, tense or high in energy.

In a separate viewing of the videos, coders rated attention similarly but on a unidimensional −100 to +100 scale, moving the joystick to the left to indicate low levels of attention to the partner’s face and to the right to indicate high levels of attention. More specifically, they gave high (more positive) scores when one individual gazed at the other’s face, intermediate scores when one individual gazed at a nonfacial body part of the other or the dyad mutually gazed at an object in the environment, and low (more negative) scores when one individual gazed away from the other. See Table 1 for summary.

**Table 1:** Operational Definitions of High/Low Levels of Affective Valence, Affective Arousal, and Attention as Continuously Coded by Trained Raters During Behavioral Coordination Task

Score	Affect		Attention	
	Valence	Arousal		
Low	Negative: sad, angry	Low energy/low intensity	One individual gazes away from other	
Medium	Neutral: neither positive nor negative	Moderate energy: alert but relaxed	Individual gazes towards nonfacial body part of other	Individual attends to object that other is also attending to
High	Positive: happy, content	High energy/high intensity	Individual gazes at other’s face	

Coders were trained on videos from three mother–infant dyads who were not included in the final sample, and during this training reached good inter‐rater reliability, ICC(2,*k*) = .7. After training, videos of mothers and infants were coded separately in an order that was counterbalanced between recorded dyads. Final inter‐rater reliability was excellent, ICC_INFANT_(2,*k*) = .877, 95% CI [.87, .88] and ICC_MOTHER_(2,*k*) = .798, 95% CI [.79, .80]. Therefore, we were able to average across the ratings of the five coders to yield six sets of data per dyad, that is, ratings of affective valence, affective arousal, and attention for both infant and mother separately, each containing 180 data points indicating ratings of affective and attentive states every s for the 3‐m analysis period.

### Plan of Analysis

To address the first aim of assessing infants’ sensitivity to bodily overlap with their mothers, infants’ looking times on the MIBO task were subjected to a repeated measures analysis of variance (ANOVA) with Stimulation (synchronous vs. asynchronous) and Identity (mother vs. stranger) as within‐participant factors. To address the second aim of investigating associations between coordination scores and infants’ preference for synchronous experience with their mother, correlations were calculated between synchrony‐preference scores (see Data Reduction) and the three behavioral coordination scores (affective valence, affective arousal, and attention) individually.

Conventional null hypothesis significance testing was used in the first instance, using a standardized alpha level of .05. Effect sizes are presented for all results; for *t*‐tests, Cohen’s *d* was used; for ANOVA results, generalized eta squared ($\eta_{G}^{2}$; Olejnik & Algina, 2003); and for correlations, nonparametric Spearman’s correlation coefficients (due to non‐normality of behavioral coordination scores). To allow us to make more meaningful inferences with regards to null results, we then conducted additional Bayesian analyses for critical tests, using JASP 0.9.0.1. The Bayesian approach offers an alternative to null hypothesis testing based on probability statements about unknown parameters (Jeffreys, 1961), and is becoming more widely used in developmental research (see Van de Schoot et al., 2014). The central benefit of the Bayesian approach to this study is that it allows us to determine whether a nonsignificant result is substantial evidence for the absence of an effect, or whether it should instead be interpreted as “inconclusive” (Dienes, 2014). For the standardized mean difference δ in the Bayesian *t*‐test, a Cauchy (0, 0.707) prior was used (Morey, Rouder, & Jamil, 2015), and for Bayesian correlational analyses, a stretched symmetric beta prior was used equivalent to a uniform prior between −1 and 1 (Ly, Verhagen, & Wagenmakers, 2016).

### Data Reduction

We initially recruited 27 dyads, six of whom were excluded from the final sample. Three dyads were removed because the infant failed to complete the required minimum number of trials (of two trials per condition) in the MIBO task, and an additional three dyads were filtered out because the data violated the assumptions of the autoregressive integrated moving average (ARIMA) modeling used to analyze the mother–infant coordination in the face‐to‐face interaction (see Behavioral Coordination section below).

#### MIBO Task

Trials having no fixations on one or both sides of the screen were excluded from the analysis (4.2% in total), resulting in a mean number of trials of 8.6 (*SD* = 2.4) per infant (a mean of 4.3 trials [*SD* = 1.4] featuring the mother, and 4.3 trials [*SD* = 1.5] featuring the stranger). The distribution of looking time scores was visually inspected, and a positive skew was evident, which is common in infant looking‐time data (Csibra, Hernik, Mascaro, & Tatone, 2016). Following Csibra et al. (2016), a log‐transform was applied but proved too strong a correction for the modest positive skew of the data, therefore a root transform was applied instead, yielding an approximately normal distribution suitable for parametric analysis. Means were calculated for each infant and each condition from these root‐transformed looking times, in the standard way by summing the number of seconds spent looking at the left and right videos across the repeated trials and then dividing each of those by the number of trials that infant received in that condition. For ease of interpretation, the results are presented in nontransformed (raw) units.

A “synchrony‐preference” score was also calculated in order to correlate each infant’s MIBO performance with performance on the behavioral coordination task. This score reflected the difference between looking times for the synchronous and asynchronous videos of the mother as a proportion of total looking time to videos of the mother (Mother_SYNCH_ − Mother_ASYNCH_)/(Mother_SYNCH_ + Mother_ASYNCH_). For comparison, the same difference score was also calculated for the stranger videos (Stranger_SYNCH_ − Stranger_ASYNCH_)/(Stranger_SYNCH_ + Stranger_ASYNCH_).

#### Behavioral Coordination

Coded behaviors were subjected to a time‐series analysis, in which coordination parameters were computed for each dyad, and each of the three behavioral domains (affective valence, affective arousal, and attention), separately. First, an ARIMA model (Gottman, 1981) was fitted to each series, using the auto.arima function in R, which implements a Bayesian inference criterion model comparison procedure that prioritizes parsimonious models by penalizing the number of parameters. The best‐fitting model was used to estimate autocorrelated components, that is, the coordination of behaviors within an individual, so that they could be removed to allow us to assess coordination *between* individuals which was our theoretical focus. Then, the residuals from the best‐fitting model were subjected to a Box‐Ljun *Q* test to ensure that they were randomly distributed. Finally, cross‐correlation functions were calculated between mother and infant time‐series. These assessed lagged correlations (i.e., correlations between one individual’s behavior and the subsequent behavior of the other individual) between the mother’s and infant’s behaviors, over and above autocorrelations.

Of the 21 dyads, we could not fit a suitable ARIMA model for one dyad for the valence and attention domains and two dyads for the arousal domain. In these cases, significant autocorrelation remained in the residuals despite attempts to partial it out, making cross‐correlation functions unreliable. Therefore, these dyads were excluded from further analysis, leaving 19 dyads for analyses of coordination in arousal and 20 dyads for analyses of coordination in valence and attention. Following Feldman (2003), we took the maximum cross‐correlation coefficient across a 7‐s lag as indicating the degree of coordination for each dyad in each domain. This “coordination score” yielded a continuous variable, with a score of 0 reflecting no correlation between the two time series, and a score of 1 reflecting perfect association. Mean coordination scores closely followed those reported in previous literature (Feldman, 2003) *M*_VALENCE_ = 0.184 (*SD* = 0.048), *M*_AROUSAL_ = 0.201 (*SD* = 0.054), *M*_ATTENTION_ = 0.205 (*SD* = 0.068).

## Results

### MIBO Task Looking Preferences

Infants had no overall preference for either the synchrony of the stimulation or the identity of the face, as shown by nonsignificant main effects of Stimulation, *F*(1, 20) = 1.02, *p* = .324, $\eta_{G}^{2}$ = .004, and Identity, *F*(1, 20) = 0.17, *p* = .681, $\eta_{G}^{2}$ = .001. Instead, infants’ preference for stimulation synchrony depended on facial identity, as revealed by a significant interaction between Stimulation and Identity, *F*(1, 20) = 7.22, *p* = .014, $\eta_{G}^{2}$ = .036. Post hoc pairwise comparisons demonstrated that when viewing their mother, infants preferred the synchronous video, *M* = 5.35 s (*SD* = 3.28) to the asynchronous one, *M* = 4.38 s (*SD* = 3.27), *t*(20) = 4.08, *p* = .0006, Cohen’s *d* = .89. When viewing the stranger, however, infants had no preference between the synchronous video, *M* = 4.57 s (*SD* = 2.89) and the asynchronous one, *M* = 4.83 s (*SD* = 3.28), *t*(20) = −1.143, *p* = .267, *d* = −.25. Finally, infants preferred to look at their mother as compared to the stranger during synchronous stroking, *t*(20) = 2.19, *p* = .040, *d* = .48, but not during asynchronous stroking, *t*(20) = 1.26, *p* = .223, *d* = −.27.

**Figure 2:**
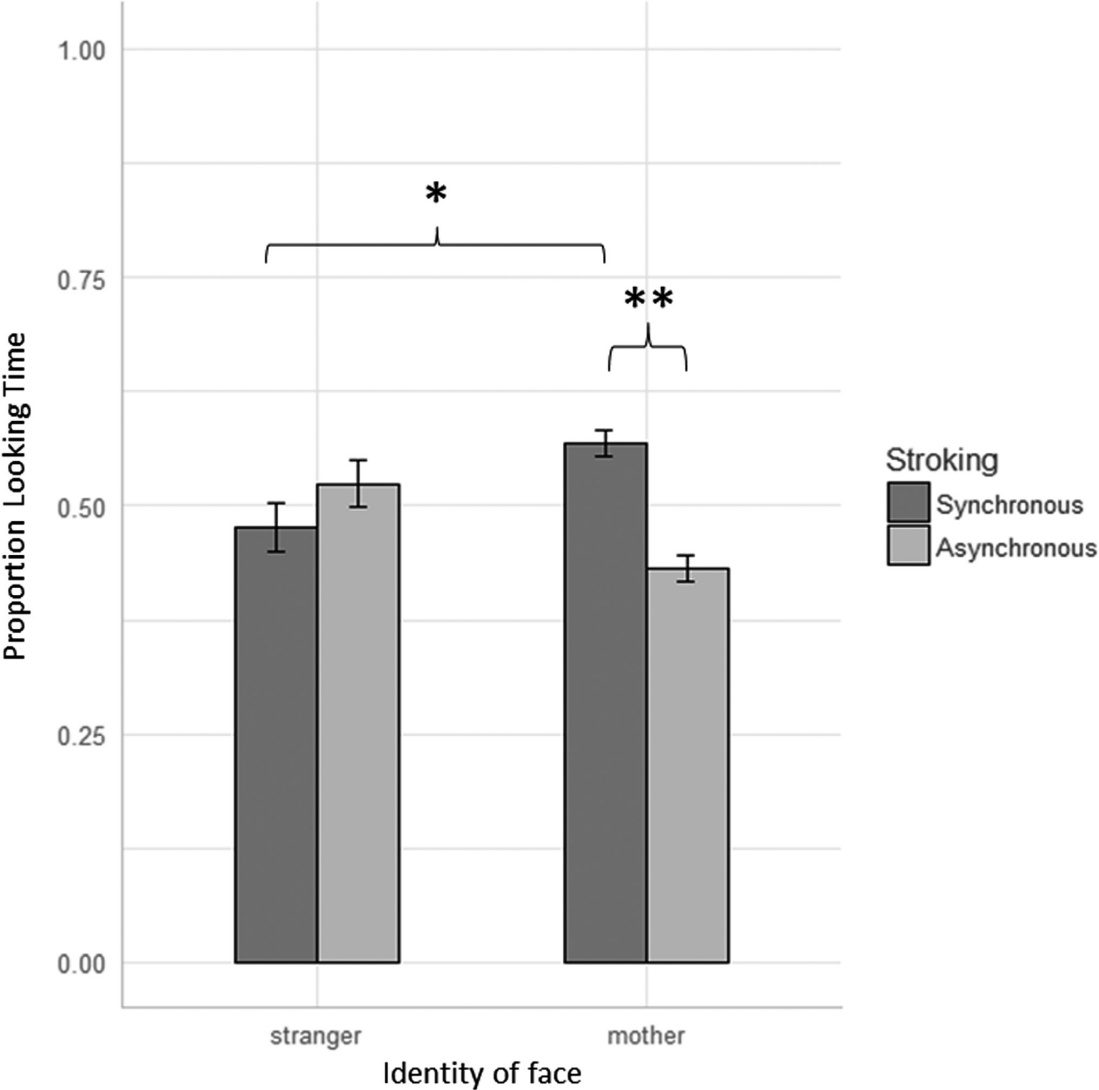
Grouped results of the Mother–Infant Bodily Overlap task (*N* = 21) showing the proportion of looking time that infants allocated to each stroking pattern when viewing the mother or the stranger. Infants had a significant preference for synchronous stimulation when observing the mother but no preference when observing an unfamiliar woman. *Significance at α < .05. **Significance at α < .01. Error bars reflect standard error of the mean.

**Figure 3:**
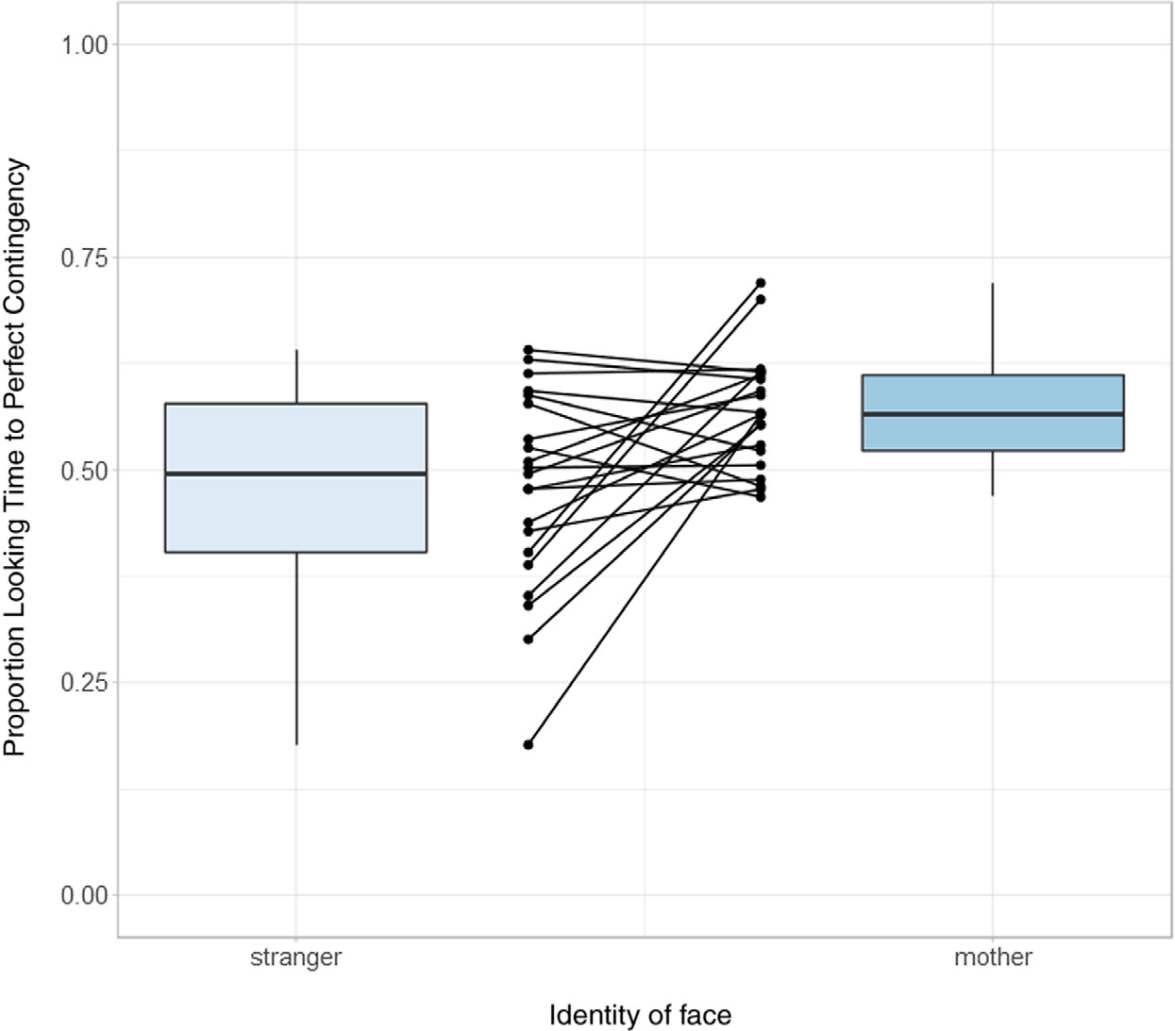
Individual results on the Mother–Infant Bodily Overlap task showing the proportion of looking time that each infant allocated to each stroking pattern when viewing the mother or the stranger. Fifteen of 21 infants (71%) had a greater preference for synchronous stimulation when observing the mother as compared to when observing an unfamiliar woman. Strip‐chart points indicate pairs of raw data points from individual infants, reflecting individual differences in looking preferences. Boxplot whiskers denote ± 1.5 × interquartile range limits. [Color figure can be viewed at wileyonlinelibrary.com]

To express the results as proportion looking times, for each infant, the looking time to the synchronous video was divided by the sum of the looking times to both videos. When looking at the mother, the mean proportion looking time to the synchronous video was 0.569 (*SD* = 0.068), and when looking at the stranger, it was 0.476 (*SD* = 0.119), which were significantly different, *t*(20) = 2.97, *p* = .007, *d* = .65. These results were also replicated using nonparametric statistics. A Wilcoxon signed rank test comparing proportion looking time to the synchronous video between mother and stranger trials confirmed that there was a significantly greater proportion looking time to synchronicity during mother trials, *V* = 183, *p* = .018. These results are illustrated in Figure 2, with data from individual infants presented in Figure 3.

To investigate whether age‐related developmental changes were related to the observed mother‐specific synchrony‐preference, the repeated measures ANOVA was then repeated with age added as a between‐subjects continuous predictor variable. The inclusion of age in the model did not alter the results. The interaction of interest, between Stimulation and Identity, remained significant, *F*(1, 19) = 7.67, *p* = .012, $\eta_{G}^{2}$ = .037. This was not modulated by age, as indicated by the nonsignificant three‐way interaction term, *F*(1, 19) = 2.25, *p* = .150, $\eta_{G}^{2}$ = .011. Furthermore, there was no main effect of age on looking times, *F*(1, 19) = 0.13, *p* = .720, $\eta_{G}^{2}$ = .005, nor did age interact with Stimulation, *F*(1, 19) = 1.25, *p* = .278, $\eta_{G}^{2}$ = .004, or Identity, *F*(1, 19) = 0.11, *p* = .739, $\eta_{G}^{2}$ = .001.

Bayesian analysis confirmed that there was decisive evidence for a synchrony‐preference when viewing the mother (Jeffreys, 1961). A Bayesian paired‐samples *t*‐test using a half‐Cauchy (0, 0.707) distributed prior comparing synchronous versus asynchronous looking times resulted in a BF_10_ = 114.99, median δ = 1.117, 95% CI [0.419, 1.813]. In contrast, when viewing the stranger, the paired‐samples *t*‐test revealed a BF_10_ = 0.12, median δ = 1.119, 95% CI [0.004, 0.446], which is substantial evidence for the null hypothesis, that is, that there is no synchrony‐preference when viewing the stranger’s face.

To ensure this effect was not due to an initial attentional preference for the mother before visual‐tactile stimulation began, an additional analysis was carried out comparing looking times to the mother and the stranger from the beginning of the trial to the onset of the first visual‐tactile contingency experienced by the infant (i.e., the first stroke on the infant’s cheek that occurred while the infant was viewing the screen). For each trial and infant, this baseline period was a minimum of 4 s in duration (as no strokes occurred in the first 4 s of any video), but was frequently longer, depending on when the infant first received a stroke while viewing the screen, *M*_MOTHER_ = 5.13 s (*SD* = 1.19), *M*_STRANGER_ = 5.32 s (*SD* = 2.31). To calculate looking times during these baseline periods, we combined the duration of looking to either side because the videos both showed the same facial identity. Baseline looking times until the first perceived visual‐tactile stimulation did not differ between trials featuring the mother versus the unfamiliar woman, *M*_MOTHER_ = 3.51 s (*SD* = 0.92), *M*_STRANGER_ = 3.49 s (*SD* = 1.29), *t*(20) = 0.08, *p* = .938, *d* = .02. Bayesian analysis confirmed this finding. A paired‐samples *t*‐test with a Cauchy (0, 0.707) prior, to test the alternative hypothesis that mother‐ and stranger‐baseline looking times were different, resulted in a BF_10_ of 0.228, median δ = 0.016, 95% CI [−0.511, 0.546], which is substantial evidence for the null hypothesis that mother‐ and stranger‐baseline looking times did not differ.

### Relation Between MIBO Task Looking Preference and Behavioral Coordination

**Figure 4:**
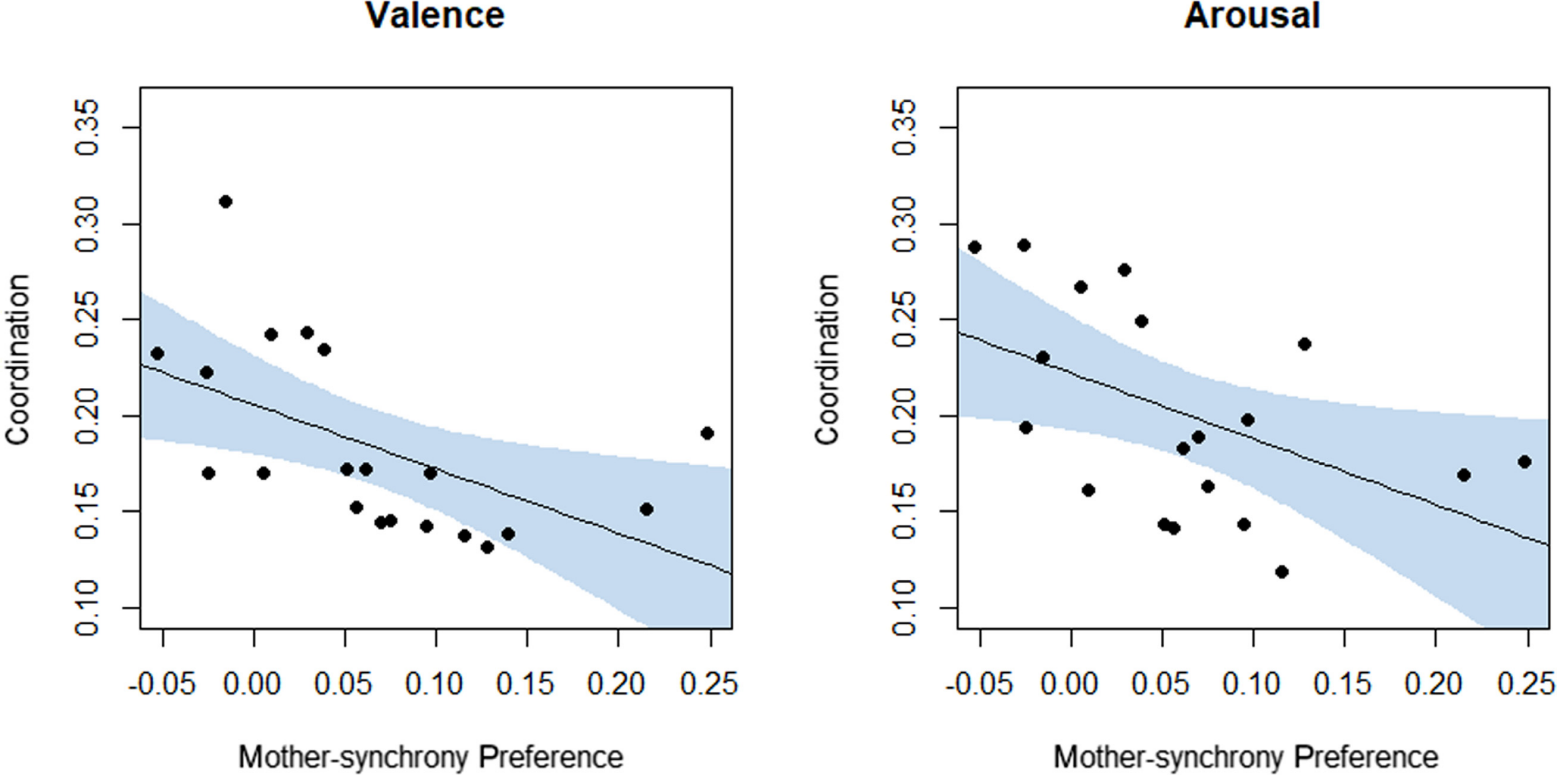
Scatter plots showing negative correlations between mother–synchrony‐preference scores and affective coordination between mother and infant during naturalistic face‐to‐face interaction. Affective coordination is separately determined as coordination of (a) valence and (b) arousal, reflecting the maximum cross‐correlation coefficient from the respective time series analyses. Mother–synchrony‐preference is expressed as a proportion difference score, reflecting looking time preference to synchronous over asynchronous visual‐tactile stimulation as a proportion of total looking time to the mother. Shaded area represents 95% confidence interval of regression estimate. [Color figure can be viewed at wileyonlinelibrary.com]

Infants who strongly preferred synchronized visual‐tactile experience with their mother had less‐coordinated affective reactions during naturalistic interactions with her. Coordination scores for both valence and arousal of affect were significantly negatively correlated with mother–synchrony‐preference; for valence, *r*(18) = −.63, *p* = .003, and for arousal, *r*(17) = −.52, *p* = .023. These results are presented in Figure 4. Interestingly, coordination scores for attention were unrelated to infants’ mother–synchrony‐preference, *r* = −.15, *p* = .539.

Further analyses confirmed that these associations were specific to a mother–*synchrony‐*preference, rather than just a more general preference toward viewing the mother irrespective of stimulation synchrony. To test this, a “mother‐preference” score was calculated. This reflected the difference between looking times to videos of the mother versus the stranger, as a proportion of total looking time to all videos ([(Mother_SYNCH_ + Mother_ASYNCH_) − (Stranger_SYNCH_ + Stranger_ASYNCH_)]/(Mother_SYNCH_ + Mother_ASYNCH_ + Stranger_SYNCH_ + Stranger_ASYNCH_)). The mother‐preference score did not significantly correlate with any of the social coordination variables; for valence, *r* = −.04, *p* = .876, for arousal, *r* = −.35, *p* = .135, and for attention, *r* = −.06, *p* = .797.

We then tested whether the association between mother–synchrony‐preference and social coordination was specific to the mother relationship, by conducting the same correlational analysis on infants’ preference for synchronous videos of the stranger. None of these correlations reached significance. For valence, *r* = −.22, *p* = .358, for arousal, *r* = −.20, *p* = .403, and for attention, *r* = .33, *p* = .158. Thus, the associations between synchrony‐preference and affective coordination appear to be specific to the infant’s relationship with the mother.

Bayesian analyses were carried out to further test the two key findings of the correlational analysis, namely the relation with valence and arousal coordination. This confirmed that there was strong evidence for a correlation between mother–synchrony‐preference and valence coordination, using a uniform prior between 1 and −1 (Jeffreys, 1961; Ly et al., 2016); BF_10_ = 4.653, 95% CI [−0.769, −0.106]. However, although there was evidence for a correlation between mother–synchrony‐preference and arousal coordination, it was only anecdotal; BF_10_ = 2.584, 95% CI [−0.750, −0.043]. In contrast, there was substantial evidence *against* the presence of a correlation between stranger–synchrony‐preference and valence, BF_10_ = 0.284, 95% CI [−0.457, 0.368]. However, although there was evidence against a correlation between stranger–synchrony‐preference and arousal coordination, it was only anecdotal; BF_10_ = 0.385, 95% CI [−0.563, 0.263]. Therefore, the Bayesian analysis provided conclusive, strong evidence for a *mother‐specific* correlation between synchrony‐preference and coordination of affective valence, but the data were inconclusive as to whether there was a similar relation with affective arousal or whether this was specific to the mother.

## Discussion

### MIBO Task Looking Preferences

In this study, we measured 6‐ to 8‐month‐old infants’ visual preferences toward perfect visual‐tactile contingencies when viewing their mother as compared to when viewing another woman. Because perfect contingencies generally indicate self‐specific stimuli, and imperfect contingencies reflect an interaction with a social object (Gergely & Watson, 1999), we interpreted a preference for perfect contingency as an indication of bodily self‐other overlap, whereby the representation of the bodily self is less‐differentiated from the representation of the other person (Maister & Tsakiris, 2015, 2016; Tsakiris, 2017).

We found that infants preferred the self‐specifying stimulation of visual‐tactile synchrony when observing their mothers, but not when observing an unfamiliar woman. Infants had no overall preference for the mother versus the stranger, so this finding was unlikely to be a general effect of attentional bias toward the mother resulting in the contingencies being more easily detectable. We argue that this mother‐specific contingency preference may indicate that there is something more “self‐like” or self‐relevant about the mother’s bodily experiences than about the experiences of unfamiliar others, when presented within the same task. In terminology borrowed from the adult literature on body representation and social interaction (see Maister & Tsakiris, 2015), there may be greater self‐other overlap between infant and mother than between infant and stranger. In more mechanistic terms, observing the mother’s body may partially activate infants’ representation of their own body, leading to enhanced self‐specific processing. Why might this be the case?

First, due to the infant’s history of being touched and stimulated by their mother, the sight of her may have activated the infant’s bodily self‐representation, as a priming of the body as a target for sensations. This may have directed the infant’s attention preferentially toward information that was self‐specific. This mechanism could function to “tune” the infants’ learning about their bodies toward information arising from their primary caregiver, allowing them to have a special influence on the development of the self (Zmyj & Marcinkowski, 2017). Parents are a relatively reliable source of information about the infant’s self‐experience due to their interaction history, so the parent’s sensations and embodied experiences are directly self‐relevant to the infant in a way that the experiences of less familiar others are not (Fonagy et al., 2007; Fotopoulou & Tsakiris, 2017).

Second, from birth, infants prefer perfect visual‐tactile contingencies only when viewing a stimulus that matches an innate, rudimentary body representation (Filippetti et al., 2013). This response is thought to reflect an early assessment of “self‐relevance,” functioning to guide their attention to self‐specifying information arising from objects in the environment that are potentially part of their body, and away from objects that are not body‐like. By 6–8 months, the age that we investigated, infants have a more well‐developed representation of their bodies in terms of what is under their direct motor control and what results in immediate double‐tactile sensation when touched (i.e., experiencing a tactile sensation synchronously both on the body part being passively touched, and the body part doing the active touching, signifying that one is touching oneself; Rochat, 2003). Therefore, it is possible that the early restriction of contingency preference to any body‐like stimuli, observed at 24 hr of age, may have become more precise for older infants, being specified more narrowly as “socially self‐relevant” when social information is available. Individuals with whom the infant has had close embodied interactions with in the past may now be perceived as more self‐relevant than strangers, leading to a preference toward self‐related sensations when in their presence.

One alternative interpretation to our claim that infants prefer self‐specifying stimulation in their mother’s presence is that our visual‐tactile stimulation was not truly perfect and therefore self‐specifying, but was instead the high‐but‐imperfect kind of stimulation that infants prefer because it signals social interaction. An experimenter cannot deliver contingent stimulation as perfectly as a mirror or a live video. The implication is that rather than experiencing perfectly contingent versus noncontingent stimulation, infants experienced imperfectly contingent versus noncontingent stimulation. We acknowledge that minor spatiotemporal discrepancies between the tactile stimulus delivered to the infant and the one delivered to the adult in the video could occur as a result of human error or infants moving their heads. However, the window of perceiving visual‐tactile simultaneity is much wider earlier in development than later, with children as old as age 9 years perceiving 1,200‐ms asynchronies as being simultaneous (Chen, Lewis, Shore, Spence, & Maurer, 2018). We contend it is thus very unlikely that infants perceived the synchronous condition as asynchronous, given that any minor discrepancies were certainly not of this magnitude. Thus, we conclude that infants were more likely to perceive our synchronous condition as perfectly rather than imperfectly contingent, and therefore self‐specifying. We also hypothesize that if we had presented infants with all three types of visual‐tactile contingencies—perfect, imperfect (social), and noncontingent—they would discriminate among all three and prefer more contingency to less. This hypothesis is based on evidence that 5‐month‐olds in a visual‐proprioceptive task look longer at imperfect contingency with an object to noncontingency (Schmuckler & Jewell, 2007) and that infants in face‐to‐face interaction studies look longer when adult mimics them than when the adult interacts either naturalistically or noncontingently (Markova & Legerstee, 2006; Striano, Henning, & Stahl, 2005).

Although we predicted that the preference for perfect contingencies would be higher when viewing the mother than the unfamiliar woman, we still expected to see an overall contingency preference across both identities. However, infants in the current study had no contingency preference when viewing the unfamiliar woman. Although contrary to our initial prediction, it is somewhat unsurprising. Previous studies demonstrating an overall preference for perfect visual‐tactile contingencies (Filippetti et al., 2013, 2016; Zmyj et al., 2011) have arguably used stimuli that already have relatively high bodily self‐relevance to the infant. Two studies utilized images of an infant face, which closely matched the infant’s own face in size and proportions (Filippetti et al., 2013, 2016). Another study used videos of infant legs, dressed in identical clothing to the infant themselves and seen from a first‐person perspective, again emphasizing the bodily self‐relevance of the visual information (Zmyj et al., 2011). Our study is the first to use two distinct social identities as stimuli, which clearly contrast in terms of their social familiarity, and do not match the infant’s body shape. We hypothesize that the video of the unfamiliar woman, presented in close temporal proximity to the video of the infant’s own mother, was perceived as less socially self‐relevant to the infant. Another possibility is that infants found the stranger so novel that they attended to processing the identity of her face at the expense of attending to differences in stimuli synchronicity. Although we cannot exclude this possibility based on the current data, it seems unlikely, because participants in similar tasks discriminated between two videos of an unfamiliar infant’s face on the basis of synchronicity (Filippetti et al., 2013, 2016). However, wariness of strangers is known to increase around this age (Sroufe, 1977), and future work can address this explanation by adding a condition with a familiarized stranger, such as a researcher who interacts with the infant before the experiment. Another way to address the possibility that the stranger triggered attentional inhibition toward the relevant information of synchronicity is to add a control condition with a non‐body‐like object such as a box being stroked synchronously versus asynchronously.

### Relation Between MIBO Task Looking Preference and Behavioral Coordination

Further emphasizing the social relevance of our findings, we found a negative correlation between mother–infant coordination in everyday interactions and the infants’ preference toward perfect contingencies when observing their mother in the MIBO task. This finding suggests that infants who experience less‐coordinated social contingencies during interactions with their mother (thought to provide the infant with essential information for self‐other differentiation) are more captured by perfect, self‐specifying information when observing her in a noninteractive context. The association found here between mother–infant coordination and mother‐specific contingency preference was present only for *affective* coordination of valence and arousal, and not for the coordination of attention to the partner’s face. This result is interesting, because it suggests that predictable coordination of affective states between infant and caregiver is related to the infant’s development of a self‐other distinction, in a way that coordination of attention is not. Indeed, it has long been argued that infants’ affective experience and regulation of their emotional states are sustained through active social engagement with others (Trevarthen & Aitken, 2001). This argument is also consistent with recent theoretical accounts that place affective intersubjectivity at the heart of an infant’s development of bodily self‐awareness (Fotopoulou & Tsakiris, 2017).

There was no association between mother–infant coordination and infants’ visual preferences when viewing an unfamiliar woman, suggesting that this result was driven by a mechanism specific to the infant–mother relationship and not a more general mechanism linking multisensory synchrony to interpersonal coordination. However, it is important to note that although the negative correlation between contingency preference and face‐to‐face coordination was specific to contingency preferences when observing the mother, we cannot make the same specificity claim regarding the face‐to‐face coordination, because we did not measure face‐to‐face coordination with an unfamiliar woman. Given this limitation, it is therefore possible that mother‐specific contingency preference may have been related to levels of face‐to‐face coordination in general, with any adult, and not necessarily coordination specifically with the infant’s mother.

The inverse correlation between bodily overlap and face‐to‐face coordination of affect is consistent with several other developmental studies. For example, 6‐month‐olds who are reported to experience more difficulties in social interaction prefer synchronous to asynchronous visual‐proprioceptive stimulation (Zmyj & Klein‐Radukic, 2015). Similarly, 3‐month‐olds who experience less coordination in naturalistic face‐to‐face interactions with their mothers later prefer to look at closely imitative, and therefore more perfect, visual‐proprioceptive contingencies when viewing the mother (Markova & Legerstee, 2006). These findings are consistent with models arguing that infants’ preference for perfect or imitative contingencies is related to less‐coordinated parent–infant interactions (e.g., Beebe et al., 2008; Gergely et al., 2010; Jaffe et al., 2001). However, the current data are the first to show a similar pattern in the visual‐tactile domain, confirming the specificity of this effect to the mother versus an unfamiliar woman, and demonstrating a significant link with affective coordination during interactions.

We speculate that higher self‐other overlap, reflected in a preference for self‐specifying contingencies when viewing the mother, mediates the link between parent–infant coordination and attachment (De Wolff & Van Ijzendoorn, 1997; Feldman, 2007; Isabella & Belsky, 1991). Although we did not directly measure attachment in our sample of infants later on, studies suggest that infants who develop an anxious, avoidant, or disorganized attachment have a history of less optimally coordinated parent–infant interactions (Isabella & Belsky, 1991; Koós & Gergely, 2001; Koulomzin et al., 2002). When these infants are in their mother’s presence, they attend more to self‐generated perfect visual‐proprioceptive contingency than to the mother’s social contingency (Koós & Gergely, 2001) and engage in more self‐touch (Koulomzin et al., 2002). Even infants who develop secure attachments prefer self‐generated perfect contingencies in their mother’s presence if she was instructed to be temporarily nonresponsive (Koós & Gergely, 2001). Our findings suggest that mother–infant interactions affect infants’ preference for perfect contingencies *only* when viewing the mother and not when viewing a stranger fits with evidence that infants who are less securely attached to their mothers engage in more self‐stimulating activities *specifically in her presence* and not that of other adults (Koulomzin et al., 2002; Main & Solomon, 1986), potentially to generate comforting experiences of causal control (Watson, 2001). A link between self‐specifying contingency preferences and attachment style is also consistent with the adult literature, which suggests that a strong drive to share contingent bodily experiences of the other within close relationships is associated with insecure attachment (Maister & Tsakiris, 2016). Although our results are correlational and therefore do not allow us to infer causality, they highlight the need for future investigations to uncover the causal mechanisms underlying these associations, potentially using a longitudinal design and mediation analysis.

### Limitations

In addition to the limitations and alternative interpretations discussed above, drawbacks of the sampling should be acknowledged. Although our a priori power analysis and Bayesian analytical method give us confidence in the interpretation of a null result, the replication of our methods on a bigger sample would further increase the reliability of our findings. The sample also lacked diversity, consisting primarily of White British families drawn from middle‐class counties, which constrains the generalizability of the findings. Infants’ bodily self‐awareness and parent–infant coordination may differ in other sociocultural contexts. Finally, infants were tested at a single time point between 6 and 8 months, which provides a snapshot of behavior in the second half of the first year, but not the developmental picture that cross‐sectional or longitudinal samples provide. Although some studies have tested 6‐, 7‐, or 8‐month‐olds (Koós & Gergely, 2001; Zmyj & Klein‐Radukic, 2015; Zmyj & Marcinkowski, 2017; Zmyj et al., 2011), others have focused on infants between birth and 5 months (Bahrick & Watson, 1985; Filippetti et al., 2013, 2016; Gergely & Watson, 1996, 1999; Jaffe et al., 2001; Rochat & Morgan, 1998). The current study replicates looking preferences found in some studies with younger infants (Filippetti et al., 2013, 2016), but does not directly capture earlier developmental features of intersubjectivity and bodily self‐awareness. Developmental changes in social referencing, joint attention, and wariness of strangers may affect infants’ behavior in both the preferential looking task and the behavioral coordination task.

### Future Directions and Open Questions

Our findings raise at least two new questions. First, why do infants prefer synchrony to asynchrony here and in other visual‐tactile tasks (Filippetti et al., 2013, 2016; Zmyj et al., 2011) when they seem to prefer noncontingency to contingency in visual‐proprioceptive tasks (Bahrick & Watson, 1985; Geangu, Benga, Stahl, & Striano, 2011; Rochat & Morgan, 1998)? On the one hand, preferences for visual‐proprioceptive noncontingency do not always replicate. Sometimes infants prefer contingency (Rochat & Morgan, 1998) and sometimes they show no preference (Geangu et al., 2011; Zmyj & Klein‐Radukic, 2015; Zmyj & Marcinkowski, 2017). On the other hand, difference in preference direction may depend on who is presented in the two preferential‐looking displays. Visual‐proprioceptive tasks address infants’ detection of self‐performed actions and thus always present the contingent self in one of the two videos. If infants recognize the contingent self as familiar, then they should prefer to look at the other video because it is novel. Visual‐tactile tasks address whether infants are sensitive to bodily overlap with another individual on the basis of synchronous stimulation. Neither video presents the self. Both present another individual receiving tactile stimulation that is either synchronous or asynchronous with the infant’s tactile experience. Because the identity of the individual is equated between videos, infants’ preferences should be driven entirely by the synchronicity of the stimulation. Infants will almost never have seen another individual experiencing perfectly contingent visual‐tactile stimulation with the self, and so they should look longer at the synchronous display than the asynchronous one because it is more novel.

A second question is whether mothers experience more bodily overlap with their own infant versus an unfamiliar infant, mirroring our finding suggesting that infants are sensitive to cues of overlap with their mother but not an unfamiliar woman. Examining overlap in both partners may establish the extent to which infants’ sensitivity to cues of overlap with their mothers is a uniquely developmental phenomenon that helps infants construct a bodily self, and not simply a defining characteristics of intimate relationships. To the best of our knowledge, no evidence to date answers this question. Although parents can recognize their own infant by sight, sound, and touch even shortly after birth (e.g., Kaitz, Lapidot, Bronner, & Eidelman, 1992) and display individual differences in their sensitivity to their infant’s bodily signals (e.g., Feldman, 2007), research on parents’ representations of their infant’s body seems scarce otherwise. New evidence on mothers’ enhanced configural processing of their own infant’s body (Montirosso, Casini, Borgatti, & Urgesi, 2016) and new frameworks on parental embodied mentalizing (Shai & Belsky, 2011) suggest this may be a fruitful direction for future research.

### Conclusions

In summary, we showed that infants preferred perfect visual‐tactile contingencies when viewing their mother, but not when viewing a stranger. Furthermore, the magnitude of infants’ preference inversely predicted mother–infant coordination in naturalistic face‐to‐face interactions. Infants who had a very strong preference for perfect contingencies when viewing the mother had less‐coordinated affective interchanges with her in routine interactions. These findings raise a number of important questions and pathways for future research in addition to those detailed above. First, in addition to replicating the findings with a larger sample size, it will be important to conduct a longitudinal study to disentangle the causal mechanisms underlying our correlational results, and enable an attachment measure to be taken in later infancy. Additional control conditions involving familiar individuals besides the mother should be included to assess whether our findings are truly specific to the mother or primary caregiver, or whether they can be more generally applied to any highly familiar individual. Comparisons between mothers and fathers might be particularly intriguing. A microanalytic approach to analysis of parent–infant interactions may yield a more fine‐grained understanding of the underlying mechanisms, as well as potential links with specific attachment patterns (Beebe et al., 2016; Isabella & Belsky, 1991). For example, would infants who experience poorly coordinated maternal interactions characterized by intrusiveness and overstimulation, predictive of avoidant attachment, experience *less* bodily overlap with their mother? Would differences in the amount of leading versus following of each partner during face‐to‐face interaction predict differences in self‐other distinction? What might be the clinical implications for infants who receive significantly less physical contact with their parents as a result of preterm birth? Future studies addressing these questions will reveal potentially fascinating mechanisms underlying our findings, and may ultimately confirm the associations among bodily self‐other overlap and dyadic coordination suggested by evidence from both infant and adult studies.
